# Itraconazole Amorphous Solid Dispersion Tablets: Formulation and Compaction Process Optimization Using Quality by Design Principles and Tools

**DOI:** 10.3390/pharmaceutics14112398

**Published:** 2022-11-07

**Authors:** Hetvi Triboandas, Kendal Pitt, Mariana Bezerra, Delphine Ach-Hubert, Walkiria Schlindwein

**Affiliations:** 1 Leicester School of Pharmacy, De Montfort University, Leicester LE1 9BH, UK; 2 Medelpharm, 615 rue du Chat Botté, ZAC des Malettes, F-01700 Beynost, France

**Keywords:** solubility enhancement, amorphous solid dispersion (ASD), hot-melt extrusion (HME), itraconazole (ITZ), tablet compaction, quality by design (QbD)

## Abstract

BCS Class II drugs, such as itraconazole (ITZ), exhibit poor solubility (1–4 ng/mL) and so require solubility enhancement. Therefore, ITZ and Kollidon^®^ VA64 (KOL) amorphous solid dispersions (ASDs) were produced using hot-melt extrusion (HME) to improve ITZ’s poor solubility. A novel strategy for tablet formulations using five inorganic salts was investigated (KCl, NaCl, KBr, KHCO_3_ and KH_2_PO_4_). These kosmotopric salts are thought to compete for water hydration near the polymer chain, hence, preventing polymer gelation and, therefore, facilitating disintegration and dissolution. Out of all the formulations, the KCl containing one demonstrated acceptable tensile strength (above 1.7 MPa), whilst providing a quick disintegration time (less than 15 min) and so was selected for further formulation development through a design of the experiment approach. Seven ITZ-KOL-ASD formulations with KCl were compacted using round and oblong punches. Round tablets were found to disintegrate under 20 min, whereas oblong tablets disintegrated within 10 min. The round tablets achieved over 80% ITZ release within 15 min, with six out of seven formulations achieving 100% ITZ release by 30 min. It was found that tablets comprising high levels of Avicel^®^ pH 102 (30%) and low levels of KCl (5%) tend to fail the disintegration target due to the strong bonding capacity of Avicel^®^ pH 102. The disintegration time and tensile strength responses were modeled to obtain design spaces (DSs) relevant to both round and oblong tablets. Within the DS, several formulations can be chosen, which meet the Quality Target Product Profile (QTPP) requirements for immediate-release round and oblong tablets and allow for flexibility to compact in different tablet shape to accommodate patients’ needs. It was concluded that the use of inorganic salts, such as KCl, is the key to producing tablets of ITZ ASDs with fast disintegration and enhanced dissolution. Overall, ITZ-KOL-ASD tablet formulations, which meet the QTPP, were achieved in this study with the aid of Quality by Design (QbD) principles for formulation and compaction process development and optimization.

## 1. Introduction

Enhancing solubility of active pharmaceutical ingredients (APIs) has always been a challenge. APIs, which belong to the biopharmaceutical classification (BCS) II, are poorly soluble and are eliminated from the body before they can fully dissolve and be absorbed [1,2]. One of the strategies to improve its solubility and, therefore, its bioavailability is to produce amorphous solid dispersions (ASDs). ASDs are molecular mixes of the drug in a hydrophilic polymer matrix [3]. As the drug exists in separate/disordered molecules in the amorphous phase, the activation energy required for dissolution in the crystalline state is avoided, thus, increasing the dissolution rate and, therefore, the solubility of the drug [4].

Hot-melt extrusion (HME) is a widely used technique to produce ASDs [5,6]. HME involves feeding the drug and polymer powders into a barrel with heating zones surrounding rotating twin screws and so the powders are mixed using heat and shear forces [7]. The resultant melt leaves the extruder from a die in the form of ASD extrudates, which are uniform in shape and density [8]. The ASDs can undergo downstream processing to finally produce tablets, capsules, granules, etc. [9]. Currently, there are 34 HME-based ASDs in the market, including an Itraconazole (ITZ) ASD for onychomycosis called Onmel^®^ [10].

Itraconazole (ITZ), a BCS Class II drug exhibiting high permeability (logP of 5.66) and low solubility in water (1–4 ng/mL), requires formulation enhancement to achieve the desired bioavailability [11]. It is an antifungal drug commercially available as an oral solution or, more commonly, as 100 mg capsules (Sporonox^®^). It has been reported that the bioavailability of the oral solution is only 55% [12]. Being a weakly basic drug, the solubility is also pH dependent, favouring acidic conditions during administration due to ionisation [7,13].

Despite the solubility enhancement of ITZ into ASDs, several challenges arise when formulating tablets of ITZ, as a high dose of 100 mg per tablet is required. The ASD form improves solubility, but the total volume occupied within a tablet formulation increases due to the presence of the polymer. ASDs allow one to achieve a supersaturated solution of poorly soluble drug and, therefore, improve bioavailability, but the polymer matrix can delay the ASD disintegration due to the polymer gelation effect [4]. During formulation development, it is also important to consider quality attributes, such as tablet size and shape, as these would ease swallowing for patients along with acceptability of the product overall.

The journey of formulation development of 30% ITZ and Kollidon^®^ VA64 ASDs (ITZ-KOL-ASD) produced using HME into tablets is explored in this paper. Through screening of tablet excipients, such as Avicel^®^ pH102 (MCC) and lactose grades, along with the use of inorganic salts, to promote disintegration was the main scope of this paper. Quality by Design (QbD) principles were also adopted using the International Council for Harmonisation (ICH) Q8 [14] and Q9 [15] guidelines. It enables a strategic product development approach, which considers process and formulation factors that may affect the critical quality attributes (CQAs) of the final product. The approach aims to achieve desired quality, increase process and manufacturing understanding and, finally, enable regulatory flexibility [16].

There is limited literature addressing ITZ ASD tablet formulations as there are several challenges due to poor compressibility and compactibility post-extrusion [17] and ASD gelation during disintegration and dissolution [18]. Zhong et al. [19] compacted ITZ and Soluplus^®^ (40% *w*/*w*) HME ASDs with 50% MCC, 42.5% croscarmellose sodium, 5% talc and 0.5% magnesium stearate, where they demonstrated a 98% release of ITZ at 10 min compared to 14% in Sporonox^®^ capsules. Previous studies have also explored the use of inorganic salts with hydroxypropyl methyl cellulose (HPMC) and Soluplus^®^ with various drugs and have found enhanced dissolution rate of the ASDs [20,21,22,23]. Takano et al. [24] also reported that tablets of ITZ ASD and potassium salts (KCl, KH_2_PO_4_, KHCO_3_) had a faster drug release compared to tablets without salts during dissolution. These are known to be kosmotropic salts, which are thought to compete for water hydration near the polymer chain, hence, preventing polymer gelation and, therefore, facilitating disintegration and dissolution [25].

In the current study, different tablet excipients (diluents and disintegrants) were initially explored to screen suitable candidates for tablets containing ITZ-KOL-ASD. Together with the ITZ-KOL-ASD and the final chosen excipients, five inorganic kosmotropic salts were then compacted: sodium chloride (NaCl), potassium chloride (KCl), potassium bicarbonate (KHCO_3_), potassium dihydrogen orthophosphate (KH_2_PO_4_) and potassium bromide (KBr). This was to determine which inorganic salts achieve tablet CQAs (tensile strength, solid fraction, disintegration and dissolution) and, therefore, meet the QTPP. The chosen inorganic salt was then formulated further into round and oblong tablets to statistically model the relationship between the amount of salt and other components (diluent) and its impact on the CQAs.

The aim of this paper was to develop novel ITZ-KOL-ASDs tablet formulations with enhanced solubility using QbD principles, which have not yet been explored previously. The objectives were, firstly, to screen suitable excipients for ITZ-KOL-ASD tablet formulations, secondly, to use mixture design to determine relationships between formulation factors and responses and, finally, to conduct statistical modeling on formulations compacted using round and oblong punches to obtain a working design space for the formulation of ITZ-KOL-ASD immediate-release tablets.

## 2. Materials and Methods

### 2.1. Materials

Itraconazole (ITZ) was purchased from Wessex Chemicals, Ashford, UK. Kollidon^®^ VA64 (KOL), Avicel^®^ pH102 (MCC) and Kollidon^®^ CL-SF (KOL-CL-SF) were donated from BASF, Ludwigshafen, Germany. Tablettose^®^ 70 (TAB70), 80 (TAB80) and 100 (TAB100) and Ludipress^®^ (LUD) were donated by Meggle GmBH & Co. KG, Wasserburg am Inn, Germany. Croscarmellose Sodium (CrNa) was donated by IMCD-DUPONT^®^, Caerphilly UK. Potassium chloride (KCl) and potassium bicarbonate (NaCO_3_) was purchased from Sigma-Merck, Taufkirchen, Germany. Potassium dihydrogen orthophosphate (KH_2_PO_4_) and potassium bromide (KBr) were purchased from Fisher Chemicals, Loughborough, UK. Standard laboratory-grade Sodium chloride was used. Magnesium stearate was purchased from Fisher Scientific, Loughborough, UK.

### 2.2. Methods

#### 2.2.1. Hot-Melt Extrusion (HME)

HME was conducted using the Nano-16 extruder (Somerville NJ, USA) with 16 mm co-rotating twin screws. The conveying screw elements were GF-A3-20-30 and kneading elements were KB7-3-15-60 F. These optimized process conditions were determined through sequential design of experiments (DoEs). A fractional factorial screening design exploring the ITZ concentration (20 to 40%), die temperature (140 to 160 °C), screw speed (200 to 400 rpm) and feed rate (7 to 9 g/min) was first conducted. The 11 runs generated using the JMP Pro 16 software were extruded. A central composite optimization study was conducted next to explore the interaction between the feed rate (5 to 9 g/min) and screw speed (200 and 400 rpm). The ITZ concentration (30%) and die temperature (150 °C) were kept constant which was determined using the first design. The working design space was obtained was used to determine the optimized extrusion processing parameters to produce 30% ITZ and KOL extrudates (ITZ-KOL-ASD) for downstream processing into tablets. The four heating zones were controlled at 120, 140, 150 and 150 °C. The feed rate was kept constant at 7 g/min using the gravimetric feeder FW20 FlexWall (Brabender Technologie, Duisburg, Germany). The screw speed was also kept constant at 300 rpm. The results for the HME process optimization will be discussed in more detail in a follow-on paper.

**Figure 1:**
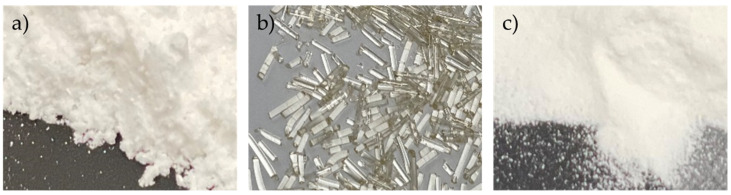
Photographs of (**a**) ITZ-KOL-PM, (**b**) ITZ-KOL-ASD extrudate pellets and (**c**) milled ITZ-KOL-ASD, for comparison purposes.

All the extrusion experiments were conducted at 18 °C controlled room temperature, with relative humidity of 41%. The extrudates were cooled using air-cooling conveyer belt (Ruhrgetriebe KG, Mülheim an der Ruhr, Germany) to allow for solidification of the extrudates. The strands were then pelletized (Accvapak System Ltd., UK) into 1 mm pellets and milled using a ball mill (Retsch, Haan, Germany) at 20 Hz in 5 min intervals for a total of 10 min. Figure 1 shows, from left to right, the ITZ and KOL physical mixture (ITZ-KOL-PM), extrudate and milled ITZ-KOL-ASD for comparison purposes.

#### 2.2.2. ITZ-KOL Amorphous Solid Dispersions

The milled 30% ITZ-KOL-ASD, with particle size between 150 and 180 μm, was used to make 100 g batches of each formulation as described in Section 2.4.5. The particle size of three inorganic salts (KCl, KHCO_3_ and NaCl) ranged from 180 to 355 μm, 180 to 250 μm for KBr and between 355 and 710 μm for KH_2_PO_4_.

The internal components (ITZ-KOL-ASD, diluents, disintegrant and inorganic salts) for the tablet formulations were mixed in 2 L plastic screw-top jars (Sunpet, Glasgow, UK) using the type T2 C turbular at 32 min^−1^ for 10 min, followed by mixing for another 5 min after the addition of the lubricant.

### 2.3. Compaction of ITZ-KOL-ASDs

Compaction was carried out using the STYL’One Nano (Medelpharm, FR) compaction simulator. Euro D round (Natoli; D = 11.28 mm) single and Euro B oblong (Natoli; 15 × 8.4 mm) punch tips were used. A V-shape profile was selected, with compaction speed of 45 mm/s. The excipients, physical mixtures (ITZ-KOL-PMs), ASD extrudate (ITZ-KOL-ASD) and tablet formulations were compacted at 50, 100, 150, 200 and 250 MPa. Final tablet formulations in the mixture design were compacted at 200 MPa.

### 2.4. Quality by Design Principles and Tools

#### 2.4.1. Quality Target Product Profile (QTPP)

First, the QTPP (Table 1) was proposed for 30% ITZ-KOL-ASD tablets with 100 mg dose, followed by identification of critical quality attributes (CQAs) of the drug product, i.e., tensile strength (>1.7 MPa), solid fraction (85 ± 0.05 %), disintegration time (<15 min) and friability <1%. This QTPP was applied for flat-faced round or convex oblong ITZ-KOL-ASD tablets.

#### 2.4.2. Identification of CQAs, CMAs and CPPs of the Tableting Process

**Figure 2:**
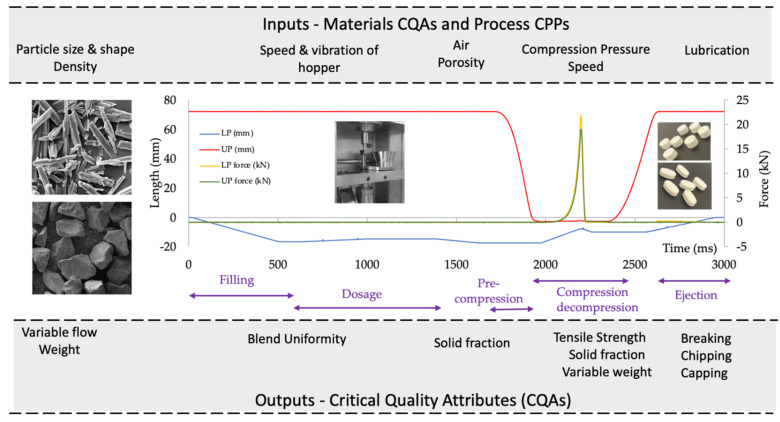
V-shaped profile (blue and red curves) indicating movements of the lower (LP) and upper punches (UP) over time and force (yellow and green curves) along in-process CQAs, material CQAs and CPPs per unit operation; pictures from left to right: SEM of crystalline ITZ (top) and milled ITZ-KOL-ASD (bottom), STYL’One nano round punch and hopper, and round and oblong tablets after compaction.

The second stage of the QbD approach involves identifying the critical quality attributes (CQAs). These are derived from the QTPP. CQAs are defined as “physical, chemical, biological or microbiological property or characteristic that should be within an appropriate limit, range, or distribution to ensure the desired product quality” [14]. In order to identify the CQAs, it is important to understand the compaction process using a V-shape profile (Figure 2).

The displacement–time profile shows filling/dosage and compression/decompression which gives the saw-toothed shape at compression speed of 45 mm/s. In the filling and dosage stages, the particles rearrange themselves until the die is filled. The calculated filling height is set to achieve a desired tablet weight of 750 mg (for a 100 mg dose). There is a linear increase in punch displacement in the next compression stage as the pressure increases, decreasing the volume within the powder and in turn increasing the powder density. In the decompression stage, the punches retreat and so there is a linear decrease in axial punch pressure until this reaches zero. Finally, the lower punch pushes the tablet out of the die which is the ejection stage [26].

Through process understanding, the potential CQAs for ITZ-KOL-ASD tablets were identified to be appearance, tensile strength, solid fraction, disintegration time, solubility and stability.

Identifying the critical process parameters (CPPs) and critical quality attributes of materials (CMAs) impacting the CQAs of the product is the next step. CPPs are “process parameter whose variability has an impact on a critical quality attribute of the product and therefore should be monitored or controlled to ensure the process produces the desired quality” [24]. The CPPs for each unit operation (filling, pre-compression, main compression, de-compression and ejection) during the direct compression process must be considered (Figure 2). CPPs include speed and vibration frequency of the hopper, compression pressure and compression speed.

CMAs are properties of the drug and excipients which should be within appropriate limits to meet desired product quality. CMAs are considered separate from CQAs as they consider the input materials rather than output materials of the intermediate/finished products [14]. CMAs for tableting include the particle size/shape, density, ITZ-KOL-ASD proportion, tablet weight, presence of air, porosity and lubrication. SEMs of crystalline ITZ and milled ITZASD are shown in Figure 2, demonstrating the difference in particle size and shape of the input and output material. Next, it is important to determine the impact of the CPPs/ CMAs on product quality in order to establish control of the CQAs [27].

#### 2.4.3. Risk Assessment

Adopting a risk-based strategy is a key science-based tool for the QbD approach and is encouraged by many regulatory agencies worldwide [28]. Risk assessment (RA) as defined in [15] is “a systematic process of organizing information to support a risk decision to be made within a risk management process”. RA aids linking the effect of CPPs/MAs on the CQAs by ranking level of risk posed. It involves three levels, risk identification, risk analysis and risk evaluation. To conduct the RA, many tools can be utilized and common tools include cause-and-effect matrix, fault tree analysis, preliminary hazard analysis and failure mode effect analysis (FMEA). FMEA was conducted in this case (Table 2).

The severity (S), occurrence (O) and detectability (D) for each failure mode were ranked. By multiplying these a risk priority number (RPN) was obtained. The severity is ranked between 1 and 10, with 1 being not severe so the CMA/CPP has no impact on the CQA and, therefore, no patient harm, whereas a ranking of 10 would be extremely severe where CMA/CPP will impact the CQA and could be life threatening to the patient. The occurrence is how frequently the failure mode occurs, with 1 being infrequent and 10 being very frequent and so affecting product quality. The detectability ranks the certainty of the failure mode being detected so 1 would mean the failure mode will certainly be detected immediately prior to downstream processing and can be controlled. A ranking of 10 would be for failure modes that cannot be detected at all during manufacture despite in-process monitoring or testing which would be very critical.

In this case an RPN less than 50 would be low risk (green), between 50 and 200 would be medium risk (amber) and more than 200 ranked as high risk (red). By conducting the FMEA it demonstrated the importance of controlling particle size and compaction CPPs (compression pressure) and CMAs (types of excipients, tablet weight and shape) as the RPN was above 200. During formulation development of ITZ-KOL-ASD tablets these parameters were taken into consideration and mitigations are suggested as shown in Table 2.

#### 2.4.4. ITZ-KOL-ASD Tablet Formulation Development

Tablets containing ITZ-KOL-ASD require a high ITZ dose of 100 mg which requires an increased tablet weight to accommodate for other excipients. Along with previous literature, an online platform called Zoomlab^TM^ [29] was utilized to explore compatibility of tablet excipients with ITZ. Zoomlab^TM^ enabled prediction of the processibility between ITZ and other excipients. The platform requires input of the QTPP and is based on the material classification system (MCS) [30]. Several material attributes were input into the online platform: the true density (1.37 g/mL) particle size (77 to 153 μm), bulk density (0.13 g/mL), tapped density (0.15 g/mL), angle of repose (66°), compaction pressure at zero porosity (1183.67 MPa), compressibility resistance (9.9), tensile strength at zero porosity (24.42 MPa) and bonding capacity of ITZ (11.56). Most attributes for ITZ were pre-filled using the platform’s database for active pharmaceutical ingredients (APIs). The next step involved selecting preferred excipients/combinations compatible with ITZ and KOL. These included Avicel^®^ pH102, DI-CAFOS A60, Flowlac, Ludipress^®^, MicroLac, Tablettose^®^ and CombiLac. A starting formulation shown by Zoomlab^TM^ with excipient blend consisting of Avicel^®^ pH102, Ludipress^®^, Kollidon-CL-SF and magnesium stearate was selected and further developed.

**Figure 3:**
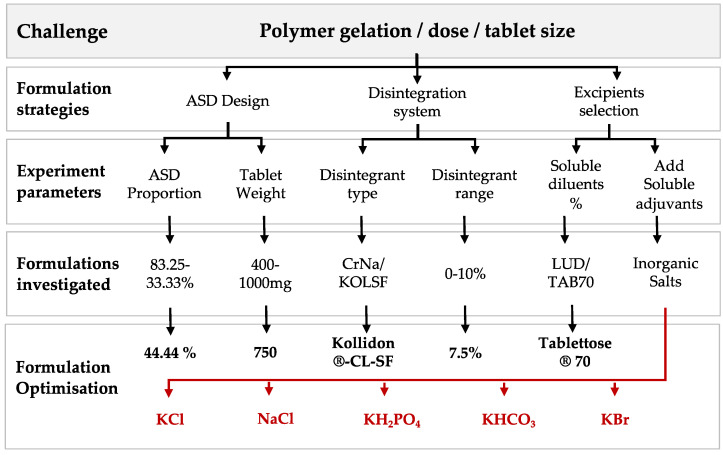
Summary of formulation development strategies.

A number of factors had to be considered when developing a tablet formulation, including the final dosage, excipient quantities and compaction behaviour and tablet weight (Figure 3).

Immediate-release tablets should disintegrate within 15 min. Hence, the first step in formulation development was exploring the disintegrant type, Kollidon^®^-CL-SF (KOL-CL-SF) and Croscarmellose Sodium (CrNa) and their amounts (4 and 7.5%).

These are disintegrants suggested by Zoomlab^TM^ [29] and reported in literature [24]. KOL-CL-SF was chosen as the primary disintegrant at 7.5% for further formulations based on this initial work.

The tablet size (400, 500, 650 and 1000 mg) and ASD proportion (83 to 44.4%) were explored next. It is well known that ASDs undergo a gelling effect due to the polymer when in contact with water [31]. The idea was to encourage disintegration by reducing the relative amount of ITZ-KOL-ASD. The 650 and 1000 mg tablets enabled disintegration as the other excipients occupied at least 50% of the tablet. A decision was made to, therefore, make 750 mg tablets containing 30% of the ASD which occupied 333 mg within the tablet to give a 100 mg dose and would enable an addition of 55.6% of soluble excipients.

Compaction of several excipients was also undertaken to determine their tabletability profile. These were Ludipress^®^ (LUD), Tablettose^®^70 (TAB70), Tablettose^®^80 (TAB80), Tablettose^®^100 (TAB100) and Avicel pH 102 (MCC.) Tablettose^®^70 was chosen to replace Ludipress^®^ as it has a narrow particle size distribution, with few fines and is ideal for ITZ-KOL-ASD tablet formulations compared to MCC. Despite a slight improvement in disintegration with formulations containing MCC and TAB70, it was very clear at this stage that a simple formulation would not provide the desired enhanced solubility due to disintegration failure.

The final strategy adopted was the addition of inorganic salts to promote disintegration. The five salts screened were sodium chloride (NaCl), potassium chloride (KCl), potassium dihydrogen orthophosphate (KH_2_PO_4_), potassium bromide (KBr) and potassium bicarbonate (KHCO_3_). Five formulations containing 44.44% ASD, 10% Avicel^®^ pH102, 27.6 % Tablettose^®^ 70, 7.5% Kollidon^®^-CL-SF, 0.5 % magnesium stearate and each salt at 10% were compacted with a tablet weight of 750 mg (Table 3). Based on the tablet-testing results KCl formulations were investigated further using design of experiments.

#### 2.4.5. Design of Experiments (DoEs)

DoEs enable statistical models to be generated which can explain the effects of the factors on the CQAs and the relationship of the input/output parameters. Through these optimal combinations of factors can be found to manufacture tablets with desired CQAs that meet the QTPP [32]. A mixture design of ITZ-KOL-ASDs was adopted. Different levels of three factors, KCl (5 to 15%), MCC (10 to 30%) and TAB70 (2.6 to 32.6%) were explored (Table 4). Seven formulation combinations were obtained using the JMP Pro 16 software; the ternary plot is shown in Table 4. The amount of disintegrant (KOL-CL-SF at 7.5%) and lubricant (magnesium stearate at 0.5%) was kept constant for each formulation.

A full compaction study was conducted from 50 to 250 MPa for formulation three with both round and oblong punches. All seven formulations were also compacted at 200 MPa into both round and oblong tablets.

### 2.5. Characterisation of Powders and Tablets

#### 2.5.1. True Density

The true density of the tablet formulations was measured using a helium pycnometer (Micromeritics, US). Sample mass of 1.6 ± 1 g was measured into a 3.5 cm³ chamber insert. The powders were first purged ten times with helium gas. The average of ten cycles was used to calculate the true density. The final true density value for each formulation (1.4 ± 1 g/ cm³) was measured in duplicates and the average was input into the Alix software. The true density was used to calculate the correct filling height for 750 mg tablets with 100 mg dose.

#### 2.5.2. USP1062 Plots

**Figure 4:**
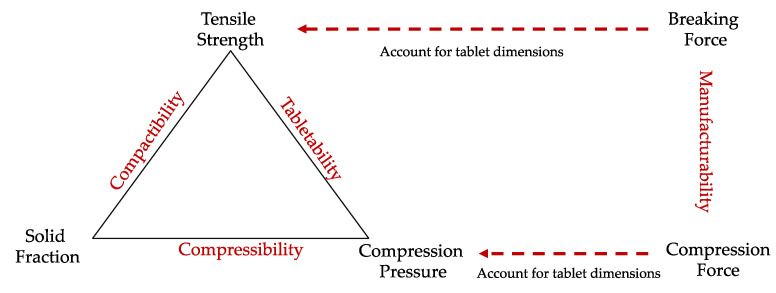
Tablet characterization summary from USP1062 guide.

It was possible to generate plots from the USP1062 guidelines [26] to analyze tablet compaction properties. The manufacturability, tabletability, compressibility and compactability plots aid manufacture and understanding of the compaction process (Figure 4).

The manufacturability is useful in a production setting as it identifies a suitable force which should be used for compression. However, the manufacturability is affected by tablet size in shape. The tabletability plot better represents mechanical strength of a tablet, as it accounts for tablet dimension by plotting compression pressure (force/area) over tensile strength (TS). The TS $\left(\sigma_{t}\right)$ equation for round- and oblong-shape tablets is shown in Equations (1) and (2), respectively, with *P* being the fracture load, *D* the diameter, *t* the thickness and *W* the tablet wall height.

(1) $$\sigma_{t}=\frac{2P}{\pi Dt}$$

(2) $$\sigma_{t}=\frac{2}{3}\left(\frac{10P}{\pi D^{2}\left(2.84\frac{t}{D}-0.126\frac{t}{W}+3.15\frac{W}{D}+0.01\right)}\right)\;\;\;\;\;$$

The compressibility shows the solid fraction (SF) of a tablet over the compression pressure. The SF is highly dependent on pressure. The SF was calculated using Equation (4). However, different parameters are considered to calculate the tablet density depending on the shape of tablet. For flat round tablets (Equation (3)), the mass is divided by pi (π) multiplied by the radius squared and thickness. For oblong convex tablets (Equation (5)) the perimeter, band width and cup volume are also taken into consideration.

(3) $$SF=\frac{Tablet\;Density\;\;}{True\;Density}$$

(4) $$Tablet\;density=\left(\frac{mass}{\left(pi*\left(radius^{2}\right)*thickness\right)}\right)\;\;\;\;\;\;$$

(5) $$Tablet\;density=\left(\frac{mass}{\left(perimeter*\left(bandwidth+cupvolume\right)\right)}\right)$$

Finally, the compactibility indicates the relationship between the TS and SF. It is important to understand the powder compaction behavior through determination of tablet porosity.

#### 2.5.3. Hardness TESTING

The hardness, weight, diameter and width measurements were recorded using a SmartTest 50 (Sotax, Foston, UK) hardness tester. Ten tablets were tested per formulation.

#### 2.5.4. Disintegration

Disintegration testing was carried out using the PharmaTest DIST3 (Pharmag, Klipphausen, Germany). The test was performed by placing six tablets in an open-ended basket which was immersed in double-distilled water at 37 °C.

#### 2.5.5. Friability

Friability testing was carried out using the PharmaTest PTF 20E D-63512 (Hainburg, DE) apparatus. Ten tablets were weighed and placed inside a rotating drum for 100 rotations at 20 rpm. Friability was calculated using Equation (6).

(6) $$Friability=\frac{\left(mass\;before\;test-mass\;after\;test\right)\;}{mass\;before\;test}\times 10\;\;\;\;\;\;\;$$

#### 2.5.6. Dissolution

Dissolution testing was carried out using an Erweka light DT 126 (Erweka Gmbh, DE) with an apparatus 2 paddle rotating at 100 rpm. A 0.1 M hydrochloric acid medium was freshly prepared using double-distilled water and maintained at 37 °C with pH1.2. Dissolution samples were collected at 1 min, 2.5 min, 5 min, 15 min, 30 min, 45 min and 60 min in 5 mL aliquots each time using emerald plastic syringes (Fisher Scientific, Loughborough, UK), passed through 0.45 µm PVDF syringe filter (Fisher Scientific, Loughborough, UK) and diluted further with 10 mL dissolution media. All diluted samples were measured at 254 nm using the Evolution 60S UV-Visible spectrophotometer (Thermo Fisher Scientific, Loughborough, UK).

#### 2.5.7. Scanning Electron Microscopy (SEM)

To characterize particle size/shape, Scanning Electron Microscope EVO LS 15 (Zeiss, Rugby, UK) was used. The powders were scattered onto the surface of aluminum stubs (6 mm height/12.5 mm disc diameter) (Agar scientific, Stansted, UK) using carbon tabs (Agar scientific, UK), followed by a layer of gold coating (15 nm thickness). The samples were measured at four magnifications (30×, 100×, 500× and 1000×).

## 3. Results

### 3.1. Characterisation of ITZ-KOL-ASD and Excipients

#### 3.1.1. Compaction of ITZ-KOL-ASDs and Their Physical Mixture

Compaction of the ITZ-KOL-ASD was conducted to initially determine compaction behavior. The physical mixture (ITZ-KOL-PM) was also investigated for comparison purposes.

**Figure 5:**
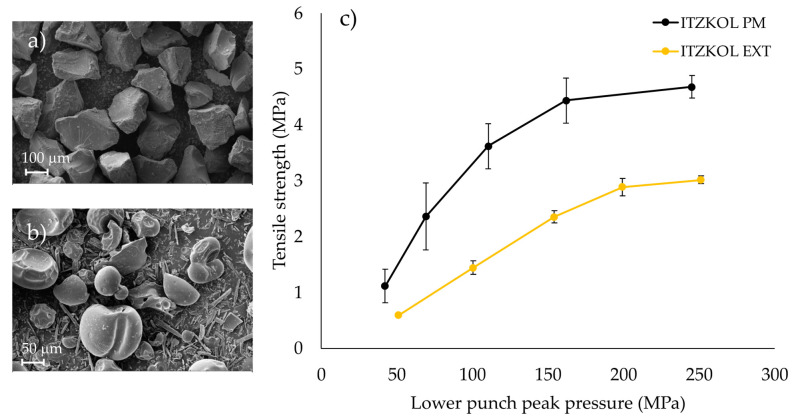
SEM pictures of (**a**) ITZ-KOL-ASD amorphous solid dispersion, (**b**) ITZ-KOL-PM physical mixture and (**c**) tabletability plots of respective ITZ-KOL-PM and ITZ-KOL-ASD tablets (*n* = 10).

The average particle size of the 30% ITZ-KOL-ASDs was between 150 and 180 μm (Figure 5a). Compared to the ITZ-KOL-PMs (Figure 5b), the ITZ-KOL-ASDs have greatly improved flowability. ITZ has a needle-like structure as a pure compound, which can be clearly distinguished in Figure 5b, as KOL can be seen to be more spherical in shape. Needle-shaped active pharmaceutical ingredients (APIs) have poor flow properties and can promote capping during tableting [30]. After extrusion, ITZ becomes amorphous within the polymer matrix and its form and size are dictated by the downstream milling process. It exhibits good flowability and so facilitates direct compression.

The tabletability plot (Figure 5c) shows the relationship between compression pressure and tensile strength. The hardness is known to be affected by several factors, including tablet size/shape, relative density, storage conditions, formulation composition and manufacturing process. Using the tensile strength and compression pressure, therefore, minimizes any variability, which could occur due to differences in tablet size, thickness and weight [26]. As the compression pressure increases the tensile strength also increases for both ITZ-KOL-ASD and ITZ-KOL-PM tablets. However, the ITZ-KOL-ASD tablets have a lower tensile strength compared to the ITZ-KOL-PM tablets in general.

#### 3.1.2. Compaction of Pure Excipients

**Figure 6:**
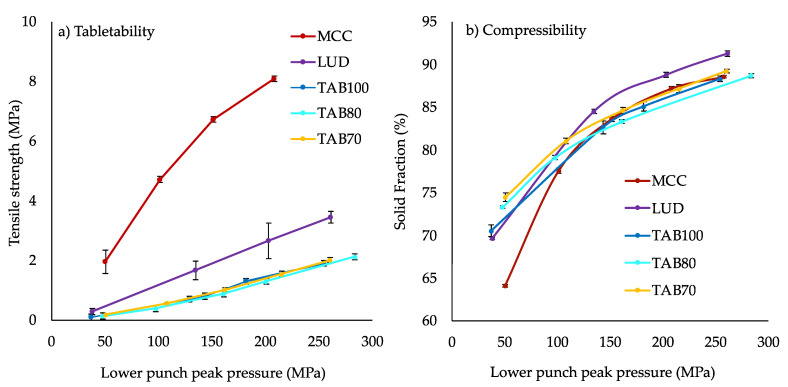
(**a**) Tabletability and (**b**) compressibility plots of excipients: MCC, LUD, TAB 70, 80 and 100 (*n* = 10).

The compaction properties of each excipient within a tablet exhibit unique mechanical properties during compaction and can also influence properties of other excipients [26]. The tabletability and compressibility plot for the excipients (MCC, LUD, TAB70, TAB80 and TAB100) explored in the formulation development of ITZ-KOL-ASD tablets is shown in Figure 6a,b, respectively. These excipients function as diluents within a tablet formulation and so enable improved flow properties and allow for a reduction in ASD percentage within the formulation, as explained in Section 2.4.4 [24]. The tensile strength for each increases as the pressure increases from 50 to 250 MPa. MCC showed a higher tensile strength (above 2 MPa) compared to the different lactose grades. The three Tablettose^®^ grades demonstrated tensile strength below 1 MPa, which is much lower than that of LUD, as LUD can also act as a binder. A lower tensile strength is associated with fast disintegration and dissolution [26]. The solid fraction of LUD was also higher (90% at 200 MPa) compared to MCC (85% at 200 MPa), producing less porous compacts, with reduced disintegration capability. Therefore, using a combination of MCC and one Tablettose^®^ grade would be ideal as it would sufficiently increase tablet strength to enable disintegration. The three TAB70, 80 and 100 have different particle size distributions: 63 to 500, 0 to 630 and 63 to 500 μm, respectively. TAB70, however, has a narrow particle size distribution (only 6% fines compared to 25% fines in TAB100) and possesses the best flow properties out of the three, with the lowest angle of repose (31°) and Carr’s Index (17.19%) [33]. TAB70 compacts have a higher solid fraction than TAB80 and TAB100 (Figure 6b). This produces stronger tablets, which have low porosity. TAB80 is recommended for low-dose formulations and TAB100 has higher compactibility properties [33]. As TAB70 has a narrow particle size distribution, with few fines, it was ideal for ITZ-KOL-ASD tablet formulations.

#### 3.1.3. Compaction and Disintegration of Formulations Containing Inorganic Salts

Despite the reduced amount of ITZ-KOL-ASD (44.4%), increased disintegrant (KOL-CL-SF at 7.5%) and the incorporation of more soluble lactose grade (TAB70), the tablets did not disintegrate. A novel strategy adding inorganic salts as soluble adjuvants within the formulation, as described in Table 3, was, therefore, introduced to promote disintegration. The compaction properties were also investigated to determine their influence on the CQAs (tensile strength, solid fraction, hardness and disintegration time).

**Figure 7:**
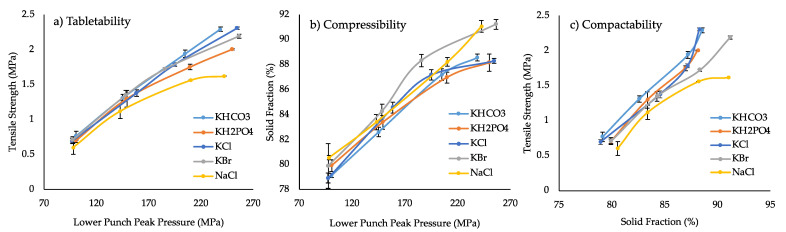
Tablets of ITZ-KOL-ASDs with inorganic salts (KHCO3, KCl, KBr, KH2PO4 and NaCl); (**a**) tabletability, (**b**) compressibility and (**c**) compactibility (n = 6).

The tabletability plot of the five inorganic salt tablets (Figure 7a) shows an increase in tensile strength with increasing pressure (linear region), which gradually levels off, despite the increase in pressure (50 to 250 MPa). This plot shows that a suitable compaction pressure within the linear region should be chosen to achieve tablets with no defects. Tensile strength above 1.7 MPa [32] is ideal to avoid breakage during storage and transport [26]. In general, a low tensile strength (less than 1.7 MPa) below compression pressure of 200 MPa can also be observed. The KCl, KH_2_PO_4_, KHCO_3_ and KBr tablets, therefore, required a higher pressure of 200 MPa to achieve a tensile strength higher than 1.7 MPa. NaCl tablets did not reach the desired tensile strength, even at high compression pressure of 250 MPa, as seen in Figure 7a.

The compressibility plot (Figure 7b) shows that all tablets containing the five inorganic salts achieved solid fraction between 80 and 90% and between 70 and 270 MPa. A solid fraction of around 85% is ideal as, below this value, tablets may be more friable and above this value capping and lamination may occur [32]. KBr and NaCl showed higher solid fraction of 88%, compared to KCl, KHCO_3_ and KH_2_PO_4_ (83%) tablets at 200 MPa. This was not expected; the NaCl tablets exhibited lower tensile strength (1.56 MPa) at 200 MPa compared to the other salt tablets.

The compactibility plot (Figure 7c) indicated a curvilinear relationship between the TS and SF. Despite producing denser tablets with solid fraction of 85%, the mechanical strength is relatively weak and does not achieve 1.7 MPa until reaching a solid fraction above 90%.

**Figure 8:**
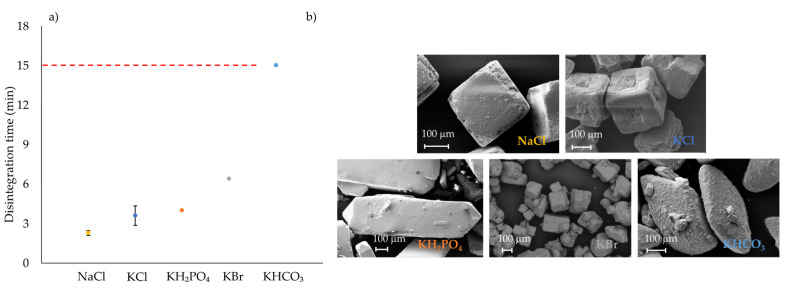
(**a**) Disintegration plot (*n* = 6) of ITZ-KOL-ASDs with NaCl (yellow), KCl (dark blue), KH2PO4 (orange), KBr (grey) and KHCO3 (light blue) salts along with disintegration limit of maximum of 15 min indicated with a red dotted line; (**b**) SEM pictures of the respective salts.

Four salts, NaCl, KCl, KH_2_PO_4_ and KBr, achieved the target disintegration time of less than 15 min (Figure 8a). NaCl disintegrated the fastest (2.29 min), followed by KCl and KH_2_PO_4_ (4 min) and finally KBr (6.4 min). NaCl demonstrated the quickest disintegration time but did not reach the desired tensile strength (1.56 MPa). The KHCO_3_ formulation disintegrated the slowest (15 min), which narrowly passes the CQA target. The SEM images of the salts are shown in Figure 8b. Out of the five salts, KCl was chosen for further investigation using a mixture design approach.

### 3.2. Formulation Mixture Design

#### 3.2.1. Compaction of Round Tablets

**Figure 9:**
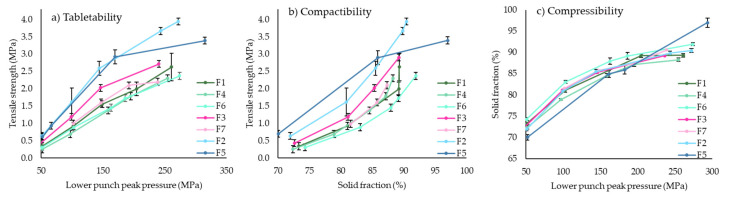
USP 1062 plots: (**a**) tabletability, (**b**) compactibility and (**c**) compressibility profiles of all round tablets (*n* = 6).

Seven formulations with different proportions of KCl were compacted to evaluate its effect on compaction. The tabletability plot (Figure 9a) shows that as the pressure increases from 50 to 250 MPa, the tensile strength of the tablets also increases and then demonstrates an independent behaviour (plateau region). Tablets with the highest level of MCC, F2 and F5 exhibited tensile strength, which surpass the target of 1.7 MPa past pressures of 100 MPa.

The dominant effect of the MCC in the formulations can be observed through the compactability plot (Figure 9b), which shows higher tensile strength (more than 2 MPa) at higher solid fraction (more than 85%). As the amount of MCC within the formulations decreases from 30 (F2/F5) to 20 (F3/ F7) to 10% (F4, F1 and F6), a mirroring decrease in tensile strength can be seen.

Another observation can be made regarding the decrease in tensile strength, as the amount of KCl within the formulations decreases from 15 to 5% (Figure 9c), at a constant MCC concentration. The opposite can be said for the solid fraction of the formulations as high KCl amounts and low MCC lead to higher solid fraction, as KCl particles are able to fill the voids in the power and reduce porosity. Overall, there is an increasing exponential relationship between the compaction pressure and solid fraction.

Considering the compaction results, tablets produced with compaction pressure of 200 MPa were chosen for disintegration and dissolution tests. Hence, further formulations were also compacted at 200 MPa only.

#### 3.2.2. Disintegration and Dissolution of ITZ-KOL-ASD Round Tablets

**Figure 10:**
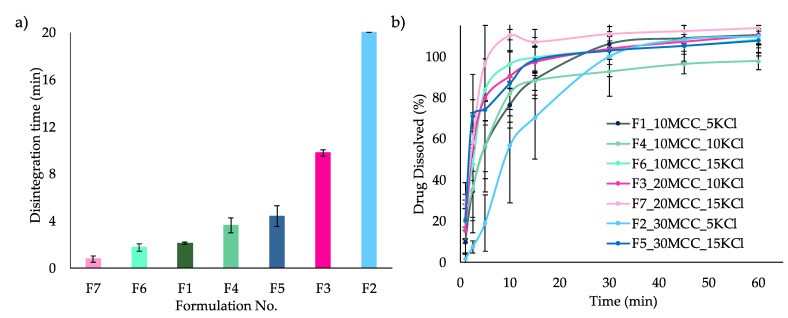
Disintegration (*n* = 6) (**a**) and (**b**) dissolution plots (*n* = 3) of ITZ-KOL-ASD round-tablet formulations.

The disintegration (Figure 10a) and dissolution (Figure 10b) behaviour of the seven formulation tablets was analysed next. The disintegration time of formulations with 10% MCC (F1, F4 and F6) ranged from 2 to 4 min, while the same response varied from around 2 to 20 min in formulations with 20–30% MCC (F2, F3, F5 and F7). These were more influenced by the KCl percentage within the formulation. For example, F7, which has 15% KCl, disintegrates in 76 s, whereas F3 takes 9.76 min to disintegrate, as it has 10% KCl. The same pattern was seen for F5 (3.39 min), which has 15% KCl and F2 (20 min) and which has only 5% KCl. Both had quicker disintegration time when KCl was increased from 5 to 15%. Comparison between 20% (F7) and 30% (F5) formulations also revealed that the MCC percentage within this high range increases disintegration time, even when both have the same proportion of KCl (15%). Overall, the disintegration time was shortest for formulations (F6 and F7) with the maximum amount of 15% KCl and MCC less than 20%, with the influence of KCl increasing with decreasing MCC content. It was also found that the presence of 10–15% KCl is likely to achieve the ITZ-KOL-ASD tablet CQAs (tensile strength above 1.7 MPa, solid fraction around 85% and disintegration time below 15 min) when compacted at 200 MPa.

Dissolution results (Figure 10b) show that the tablets achieved over 80% of ITZ dissolved within 30 min and most of the tablets, except F4, reached 100% at 60 min. The highest C_max_ was obtained with tablets containing 15% KCl after 15 min (F5, F6 and F7). A very quick release followed by an immediate decrease was also observed for F7. The delayed profile of F2 (30% MCC and 5% KCl) was also seen at 20 min, as reflected by the disintegration time of around 20 min.

The disintegration order from quickest to slowest was F7, F6, F1, F4, F5, F3 and F2, whereas at 15 min, the dissolution order varied slightly with F1, F6 being quickest again followed by F5, F3, F1, F6 and, again, F2 being the slowest. Despite F4 disintegrating within 5 min, it takes just over 30 min to achieve 100% ITZ dissolution compared to the rest of the formulations. These manage to achieve 100% drug dissolution before 30 min. Once again, KCl has a higher effect on dissolution with low MCC (10%) content but by 30 min, 100% drug release is still achieved. F4, with equal amounts of KCl (10%) and MCC (10%), had the poorest dissolution performance, as it also exhibits the lowest tensile strength, as shown in the plot in Figure 9a.

### 3.3. Round Versus Oblong ITZASD Tablets

#### 3.3.1. Compaction Study

A study was also conducted to determine the impact of different tablet shapes on compaction and disintegration ability of the same formulations. As 750 mg round tablets are bigger than standard tablets, it was important to assess the ease of interchanging tablet shapes during manufacture based on patient acceptability. For this purpose, compaction and disintegration behaviours of round- and oblong-shaped tablets were compared using the F3 formulation (20% MCC, 10% KCl and 27.6% TAB70).

**Figure 11:**
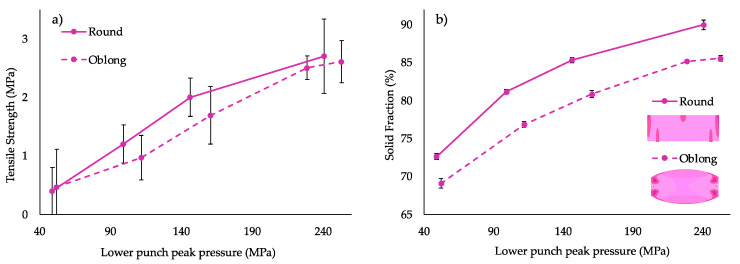
(**a**) Tabletability plot for round/oblong ITZASD tablets with the TS $\left(\sigma_{t}\right)$ and (**b**) compressibility plot and cross-sections of high- and low-density distribution for round/oblong ITZASD tablets, respectively (*n* = 5).

The five-fold increase (50 to 250 MPa) in compression pressure increases tensile strength considerably for both tablet types (Figure 11a). In general, it was found that the tensile strength was slightly lower for oblong tablets compared to round tablets of the midpoint formulation (F3). As the difference in tensile strength was small for both ITZ-KOL-ASD tablets (2.5 MPa for round and 2.7 MPa for oblong at 200 MPa), the results are quite comparable. HME ASD tablets are known to require high compression pressures to achieve tensile strength above 1.7 MPa [13]. It is also important to consider the differences in the calculation for tensile strength of flat round (Equation (1)) and elongated (oblong) convex-shaped tablets (Equation (2)). For elongated tablets, increasing the length-to-diameter ratio results in limiting the stress value to 2/3 of the round tablets [34]. The tensile strength values should agree with each other, which was the case for higher compression pressures (200 MPa).

The compressibility plot (Figure 11b) also demonstrated similar solid fraction behavior between round and oblong tablets, with the oblong having a lower solid fraction. The solid fraction is calculated by dividing the tablet density over the true density for standard round flat tablets (Equation (4)) [13]. However, for oblong tablets, further considerations, such as the perimeter, band width and cup volumes, must be considered (Equation (5)). As density is a man factor to calculate the solid fraction, differences in the density distributions between flat planer and convex tablets should be noted here. It has been reported that curved tablets have high-density regions on the edges of the tablets (Figure 11b bottom image), whereas flat tablets have high-density regions in the top corners and in the center of the bottom half (Figure 11b top image). There is a lateral movement during compression in the higher-density regions, which form areas that are softer and, therefore, can be separated easily [35]. This is more of an issue with curved tablets, as particles tend to move to the center of the tablet, giving greater variation in density compared to flat-face tablets, which may be contributing to a lower solid fraction in the oblong tablets. This, however, has not been explored extensively previously for oblong tablets.

For both sets of tablets, compression pressures above 150 MPa are required to achieve solid fraction between 80 and 90%. To achieve tensile strength sufficiently above 1.7 MPa and solid above 85%, compression pressure of 200 MPa was, therefore, selected for the compaction of the seven mixture ITZ-KOL-ASD formulations.

#### 3.3.2. Modelling the Effects of MAs on CQAs

**Figure 12:**
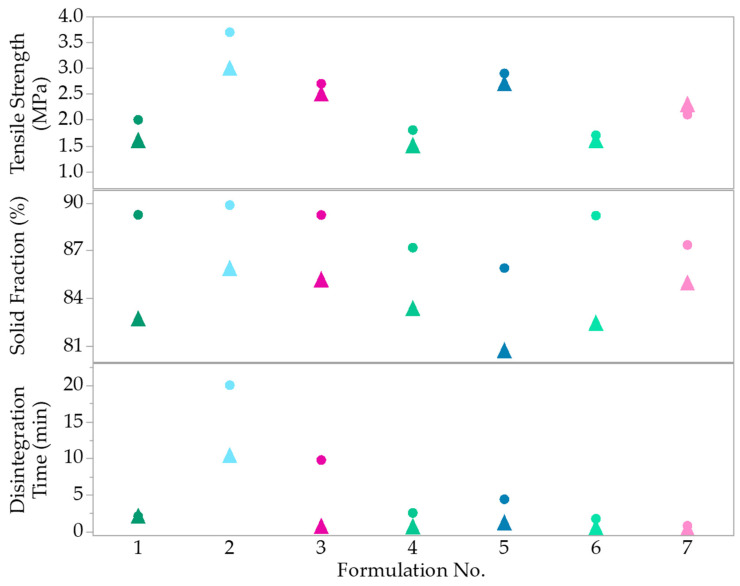
ITZ-KOL-ASD round (dots) and oblong (triangles) tablet formulations (F1 to F7) against CQAs measured.

The factors and responses (CQAs) of tablets manufactured at 200 MPa, for both round and oblong tablets, are shown in Table 5, along with a visual representation of the results in Figure 12. Oblong tablets using the same mixture design (Table 4 in Section 2.4.5) were compacted to also compare the influence of tablet shape on the CQAs.

For the tensile strength response, both round and oblong tablet formulations with the highest amount of MCC (30%) exhibit the highest tensile strength (2.5 to 3.7 MPa), followed by 20% MCC. Formulations with 10% MCC have tensile strength below 1.7. These formulations would not pass the QTPP. The solid fraction of the round tablets passes the QTPP (85 ± 0.05%), with round tablets also exhibiting slightly higher solid fraction for each corresponding formulation. For the oblong tablets, all formulations, except F5, meet the target solid fraction range. F5 can still be counted as borderline pass, as it comfortably meets the tensile strength (2.7 MPa) and disintegration time (1.16 s) target.

For the round tablets, six out of seven tablets passed the disintegration target (less than 15 min). F2 with the highest amount of MCC (30%) took 20 min to disintegrate, which does not meet the QTPP. Oblong tablets disintegrate much quicker compared to the corresponding round tablets (Figure 12). Most oblong tablet formulations disintegrated within two minutes, in comparison to the round tablets. The oblong shape improved disintegration, even for formulations with 30% MCC (F2 and F5), with all formulations meeting the disintegration target of less than 15 min. Using the tensile strength and disintegration time responses, statistical modelling was conducted.

**Figure 13:**
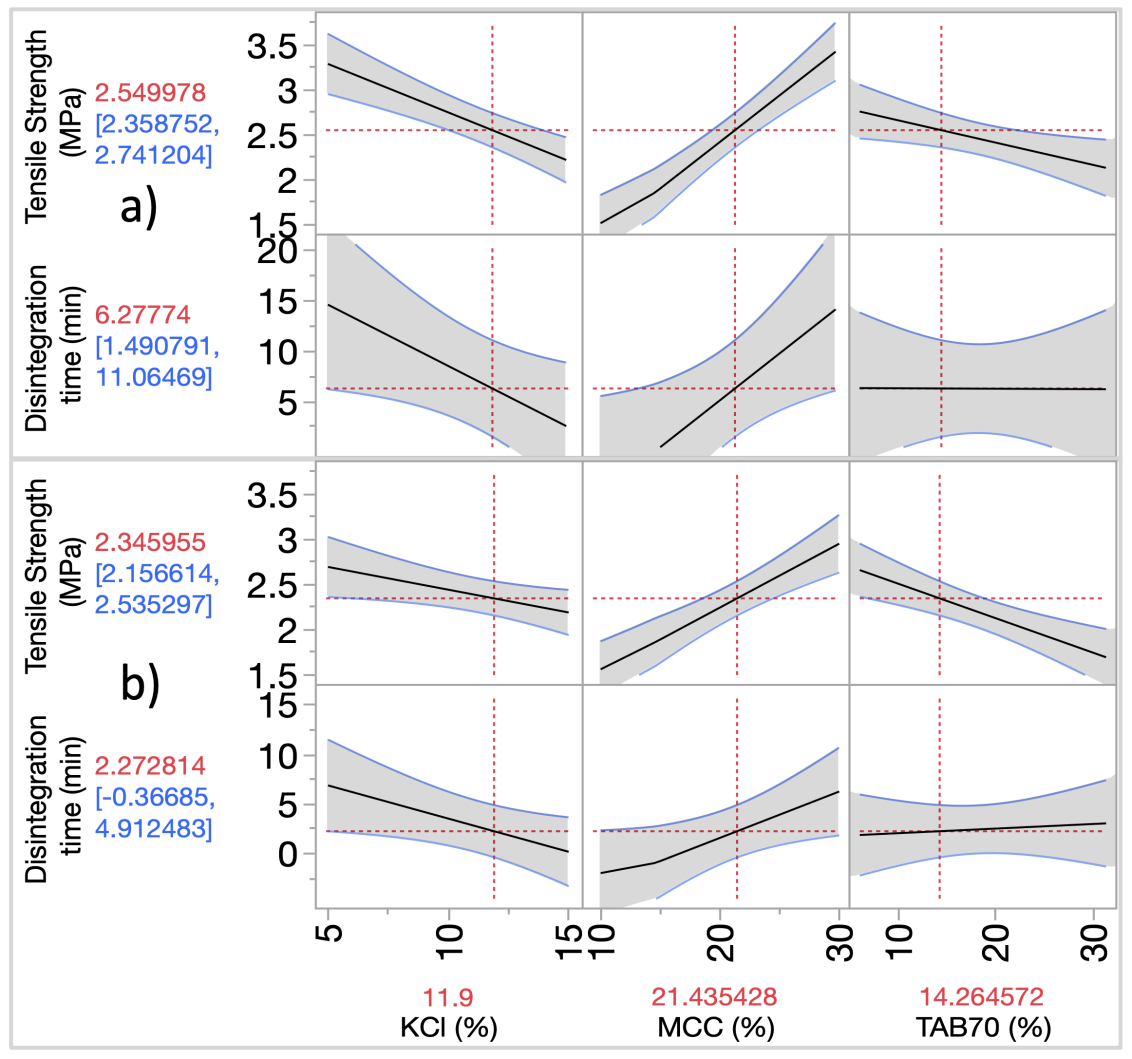
Prediction profiler for the 3 factors (KCl, MCC, TAB70 %) and responses measured (disintegration time (min) and tensile strength (MPa) for (**a**) round and (**b**) oblong ITZ-KOL-ASD tablets.

Using the prediction profiler (Figure 13), the impact of the three factors on the responses can be simultaneously visualized [36]. The correlations between the KCl, MCC and TAB70 and the responses for both round (a) and oblong (b) have the same behaviour, showing similarity between the models. The proportion of MCC has a positive effect on the tablet tensile strength, whereas TAB70 shows a negative relationship with the same response. The components are dependent, so the TAB70 effect could be explained by the lack of MCC. Disintegration time is influenced by both KCl % and MCC %. The steeper lines indicate a greater effect of the MCC and KCl on the TS and disintegration time. MCC has a dominant effect until higher levels of KCl (15%) are present, which lower the tensile strength (3 to 1.6 MPa). TAB70 only seems to impact the tensile strength compared to the disintegration time (flat line).

The R^2^ adjusted (Table 6) explained around 90% variation for the tensile strength response of both the tablets. For the disintegration time response, only 60% of the variation was explained for both models. Smaller confidence intervals (grey-shaded region) equate to higher signal-to-noise ratio (F ratio). Only the F ratio for tensile strength for both tablets (53.23 and 38.23) was statistically significant, as the probability was less than 0.05 (Table 6) [36]. Even though the F ratio for the disintegration responses was round 6, the model is still useful to understand the influence of the factors on disintegration time. F ratio above 1 means that the variability explained by the model is “sufficiently” higher than the dataset variation estimated to be due to pure error [36].

Two sets of design space were then established using these two responses in order to obtain a working design to produce ITZ-KOL-ASD tablets, which would pass for both round and oblong shapes.

#### 3.3.3. Design Space

The objective of building the statistical model was to estimate effects of the different formulation components on the tablet CQAs. The CQA targets were set to provide tensile strength above 1.7 MPa and disintegration time below 15 min.

**Figure 14:**
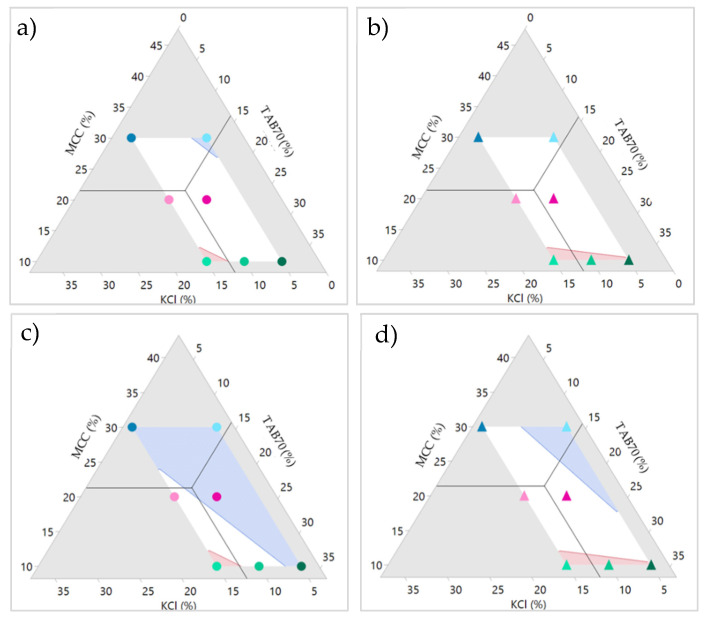
Design spaces generated for round (dots; **a** and **c**) and oblong (triangle; **b** and **d**) ITZASD tablets with tensile strength target more than 1.7 MPa and disintegration target of 15 min for (**a**) and (**b**) and 5 min for (**c**) and (**d**); marker at verification formulation with 11.9% KCl, 21.43% MCC and 14.26% TAB70.

The design spaces for the round and oblong tablets are shown in Figure 14a,b. The ternary diagram white region shows that the combinations of MCC, TAB70 and KCl mixed with the other fixed excipients (Table 4) and compacted at 200 MPa will produce tablets with targeted CQAs. The red (tensile strength) and blue (disintegration time) shaded areas represent the limits of the CQAs. Beyond these regions, formulations with ITZ-KOL-ASD will not achieve the desired QTPP. Along with formulations F3, F5 and F7, within the white region, any formulation combination will achieve the CQAs. The design space for both the round and oblong tablets is similar and so it would be possible to select one or multiple optimised formulations within this to verify the model. For example, the current value shown in Figure 14a,b, using the black lines, marks a verification formulation (V1) with 11.9% KCl, 21.43% MCC and 14.26% TAB70, which, in theory, will pass the QTPP requirements.

Along with flexibility of choosing a formulation in the design space, the quality targets can also be tightened to only give formulations, which will, e.g., disintegrate within 5 min, but will still achieve tensile strength of more than 1.7 MPa (Figure 14c,d). This would mean the V1 formulation may fail if compacted as a round flat tablet. This can be seen in Figure 13, as the disintegration time was predicted to be 6 min. Therefore, other formulations in the white regions in Figure 14c should be considered if these conditions are chosen.

Overall, if the minimum targets of 1.7 MPa and 15 min are used, there are more formulation options to compact using either round or oblong punches, which will achieve the desired CQAs. As round and oblong results were very similar, a final study was conducted to compare the dissolution performance of just the round tablets, with a verification (V1) formulation containing 11.9% KCl, 21.43% MCC and 14.26% TAB70 compacted with KCl (ITZ-KOL-ASD plus KCl) and without KCl (ITZ-KOL-ASD minus KCl) and also with a physical mixture formulation containing KCl (ITZ-KOLPM plus KCl).

### 3.4. Dissolution Study of ITZ-KOL-ASD Round Tablets

The dissolution performance of V1 formulation (ITZ-KOL-ASD plus KCl) chosen from the design space was assessed (11.9% KCl, 21.43% MCC and 14.26% TAB70). Along with this, two formulations were further compacted using the round punch at 200 MPa and a dissolution test was also conducted. Firstly, one formulation contained no KCl (ITZ-KOL-ASD minus KCl) to enforce whether the addition of inorganic salts enhances dissolution. This contained 20% MCC and 27.6% TAB70, along with identical proportions of ITZ-KOL-ASD (44%), KOL-CL-SF (7.5%) and MgSt (0.5%) as the V1 formulation. The other formulation that was compared contained a 30% crystalline ITZ and 70% KOL mixture instead of the ITZ-KOL-ASD, along with KCl and the other components identical to the V1 formulation (ITZ-KOL-PM plus KCl).

**Figure 15:**
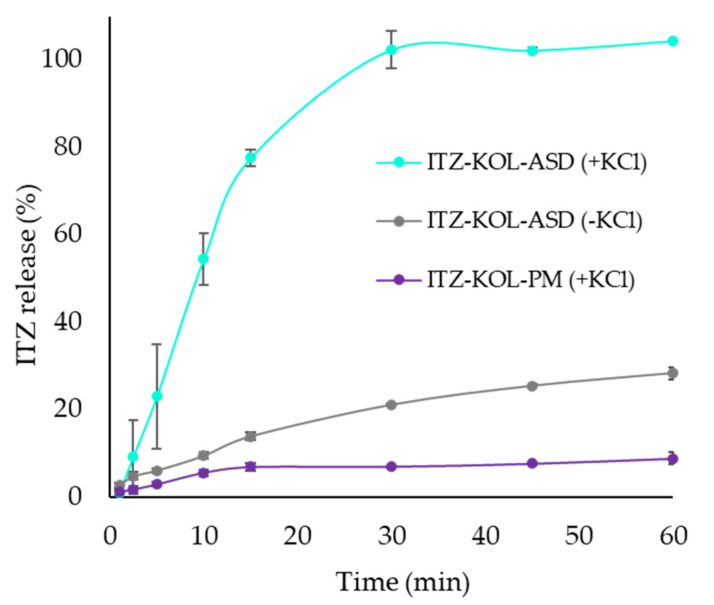
Dissolution profile for tablets (*n* = 3) of ITZ-KOL-ASD(±KCl) and ITZ-KOL-PM(+KCl).

Comparing formulations of ITZ-KOL-ASD plus KCl and ITZ-KOL-ASD minus KCl (Figure 15), the dissolution profiles that demonstrated ASD formulations provided a much “higher” ITZ release with 100% release at 30 min, compared to only 7% release at 30 min. This shows that the ASD has better solubility compared to crystalline ITZ. Comparing ITZ-KOL-ASD formulations, with (100% drug release) and without KCl (21% drug release), there is a five-fold increase in dissolution overall. This demonstrated the large impact of KCl on the release of amorphous ITZ. The addition of KCl, therefore, plays a crucial role in dissolution and, in turn, the bioavailability enhancement of ASD tablet formulations.

Other CQAs were also measured to understand effects of ASD and KCl in the tablet (Table 7). The tensile strength decreases with the addition of ASD (4.4 to 2 MPa) and KCl (3.2 to 2 MPa). The solid fraction remains around 90 ± 1 % and friability values lie below 1 (0.23 to 0.26%) for all three tablets. Considering all the CQAs, the V1 formulation (containing ITZ-KOL-ASD and KCl), which was obtained from the design space (Figure 14a), passed all the targets to achieve the QTPP for ITZ-KOL-ASD tablets with enhanced solubility.

## 4. Discussion

It was found that ITZ-KOL-ASD tablets exhibit much lower tensile strength compared with their corresponding crystalline ITZ and KOL physical mixture (ITZ-KOL-PM) tablets. None of these tablets were successful in promoting disintegration. As ITZ is poorly soluble, a tablet of the physical mixture is bound to not disintegrate. ASDs improve solubility; however, in the presence of 70% polymer, a gelation effect can occur. This is where the polymer network causes a delay in water intake and so slows down drug release [5,18]. The addition of diluents and disintegrants is, therefore, required to facilitate disintegration and dissolution of the ASD [37].

Diluents with similar functionalities might have different material attributes that can impact tablet CQAs, such a tensile strength. Diluents form the backbone of tablets, as they fill the gaps between the ASD particles [37]. MCC is one of the most common diluents used in tablets, as it enables the production of tablets with high mechanical strength at lower forces [38]. This is because MCC is a soft ductile material, which undergoes plastic deformation during the compression stage [39]. This leads to a change in shape and so increasing contact area between particles, which contributes to tablets with high compact strength and low porosity [26]. However, very high tensile strength and solid fraction are not ideal. In the initial stages of formulation development for ITZ-KOL-ASD tablets with just 12.5% MCC, 4% disintegrant (KOL-CL-SF and CrNa) and 0.5% MgSt were compacted at 200 MPa. The tablet weight selected was 400 mg, which contained 83.25% ITZ-KOL-ASD. However, formulations of both types of disintegrant failed the disintegration test for immediate-release ITZ-KOL-ASD tablets. It was evident that a diluent, such as lactose, which is more soluble and brittle in nature was also required [40] to balance the high compactibility.

LUD is composed of Lactose monohydrate (93%), Kollidon^®^ 30 (3.5%) and Kollidon^®^ CL (3.5%). The presence of Kollidon^®^ 30 with MCC contributes to higher tensile strength during direct compression [41]. This would have a counter-effect on ITZ-KOL-ASD formulations by forming harder tablets with high tensile strengths. The Tablettose^®^ ranges are ideal candidates for ASD formulations, as these are agglomerated alpha-lactose monohydrates, which exhibit good flowability and compactibility [33].

Despite exploring several formulation strategies to promote disintegration of ITZ-KOL-ASD tablets, including increasing tablet weight to 750 mg, decreasing ASD percentages below 50% to reduce polymer gelation effects makes it evident that these strategies were not fulfilling the QTPP, as disintegration failed.

The addition of five inorganic salts, NaCl, KCl, KH_2_PO_4_, KHCO_3_ and KBr, facilitated disintegration. These are also known as kosmotropic salts and so compete for water to prevent polymer gelation, hence, facilitating disintegration [42]. The anions Cl^−^, PO4^−^, CO_3_^2−^ and Br^−^ have large negative Gibbs free energy in hydration, as they are kosmotropic and so have a structured water ‘lattice’ around the ion [42]. The salt prevents gelling of the polymer in the ASD by competing for water instead (salting-out effect) [24]. Interestingly, the anion efficiency did not strictly follow the proposed Hofmeister series for kosmotropic salts (CO_3_^2−^ > PO_4_^−^ > Cl^−^); similar findings have been reported in the literature [43]. These anions cause modifications between the salt–polymer interactions, modifying the structure of water by the presence of ions, which impacted the disintegration mechanism, therefore, allowing for disintegration of ITZ ASD tablets [31]. This salting-out effect to improve disintegration has been previously found to be 20% higher with the addition of 6–10% kosmotropic salts. 

Overall, the current findings are also in agreement with the addition of inorganic salts accelerating disintegration significantly.

KCl, KH_2_PO_4_ and KBr tablets achieved the desirable tensile strength (above 1.7 MPa at 200 MPa) whilst providing a quick disintegration time. All inorganic salt tablets achieved friability below 0.53%, which complies with the proposed QTPP. Considering the compression properties, disintegration results and particle size and shape, the KCl salt was, therefore, chosen as the model salt for further formulation development using a mixture design approach.

After determining formulation components, which would prompt disintegration in ITZ-KOL-ASD tablets, another study was conducted to evaluate the effects of ITZ-KOL-ASD composition, in other words, proportions of two diluents (MCC and TAB70) and KCl on the CQAs, to finally find an optimized formulation. Seven formulations of the mixture design were compacted into round and oblong tablets to also determine if a flexible approach to tablet manufacturing can be adopted.

It was found that MCC had the greatest influence on tensile strength and disintegration time of both round and oblong tablets. It was expected that MCC would have a considerable impact on the tensile strength. The MCC effect on disintegration could be connected to excipient wetting/disintegration mechanism and/or the level of densification provided to the tablet structure [44]. Fracture planes in the cellulose chains have also been reported to occur on the surface rather than through the chains, which leads to stronger bonding area, consequently reaching zero porosity at high compression pressures of 200 MPa [45]. The presence of KCl within the formulations decreased tensile strength and improved disintegration.

Compared to round tablets, oblong tablets exhibited lower solid fraction and tensile strength. This observation can be hypothesised to be also due to different length-to-diameter ratios and varying density distributions of oblong tablets, compared to round flat-faced tablets. This behaviour is also reflected in the disintegration results. The disintegration time decreases dramatically for oblong tablets (10 min) compared to round tablets (20 min), which contain the highest amount of MCC (30%), as MCC has higher capacity to form stronger bonds [26].

To further enforce the role of KCl for ITZ-KOL-ASD tablets, a final study was conducted to compare in vitro dissolution of the optimized formulation (V1; 11.9% KCl, 21.43% MCC and 14.26% TAB70) with no KCl, along with replacing the ITZ-KOL-ASD with the crystalline ITZ and KOL mixture. A five-fold increase in dissolution was found for the optimized-formulation (V1) tablets, where 100% ITZ release was seen at 30 min, compared 21% ITZ release for the tablets with no KCL. The ITZ-KOL-PM (with KCl) tablets could only achieve 10% ITZ release over 60 min. This proved that ASDs improved solubility of ITZ, but inorganic salts, such as KCl, are equally important to enhance the disintegration, dissolution and overall solubility of ITZ-KOL-ASD tablets.

## 5. Conclusions

In this paper, 30% ITZ-KOL-ASDs, manufactured using HME, were used for downstream processing into tablets by adopting QbD principles. A QTPP was proposed and several formulation strategies were adopted to overcome the poor disintegration and dissolution CQAs of ITZ ASD tablets. In the preliminary formulation stages, it was found that disintegration was not possible solely by decreasing the polymer amount, increasing disintegrant (7.5%) and adding soluble diluents (TAB70). The incorporation of five inorganic salts (KCl, NaCl, KH_2_PO_4,_ KHCO_3_ and KBr) greatly facilitated and improved the disintegration of ITZASD tablets. It was found that three inorganic salts, KCl, KH_2_PO_4_ and KBr, possessed the ability to achieve the desired CQAs and pass the QTPP requirements for immediate-release ITZ tablets.

A second study was conducted to find an optimized tablet formulation of round and oblong ITZ-KOL-ASD by exploring MCC, TAB70 and KCl proportions, through the mixture design approach. MCC had a dominant effect on the formulation at higher levels of 30%. By incorporating 10–15% KCl, with less than 20% MCC, tablets were able to disintegrate within 4 min. It was also found that tablets produced using the oblong punches exhibit slightly lower tensile strength and solid fraction compared to round-flat tablets. Statistical modelling using JMP also supported the observations as the F ratio for tensile strength of round (54%) and oblong (38%) tablets were statistically higher compared to the disintegration time (6%). Overall, it was possible to obtain a design space relevant to both round and oblong tablets, within which several different formulations can be chosen that would meet the CQAs (disintegration within 15 min and tensile strength 1.7 MPa) and will allow for flexibility to compact in different tablet shapes to accommodate patients’ needs.

The dissolution profiles showed that the ASD formulation, in general, demonstrated a better ITZ release compared to its crystalline form (7 to 21%). The greatest enhancement in dissolution was achieved through the incorporation of the KCl in the ASD formulation, 20 to 100% release of amorphous ITZ at 30 min. Through the dissolution study, it can be concluded that inorganic salts are the key to producing novel tablets of ITZ-KOL-ASDs with fast disintegration and dissolution. This study, overall, demonstrated formulation strategies to address poor solubility of ITZ-KOL-ASD tablets while implementing QbD principles for the development and manufacture of ASD tablets.

## Figures and Tables

**Table 1:** Quality Target Product Profile of ITZ-KOL-ASD tablets.

Quality Attributes	Target
Intended use	Antifungal medication
ITZ-KOL-ASD ^1^	Itraconazole amorphous solid dispersion with 150 < PSD < 180 μm
Dosage strength	100 mg
Dosage form	Uncoated tablets
Route	Oral administration
Appearance	Flat faced round or convex oblong tablets containing ITZ-KOL-ASD
Dosage weight	750 mg ± 5%
Assay	90–110%
Solubility	>80% in 15 min
Disintegration time	<15 min
Friability	<1%
Tensile strength	>1.7 MPa (target 2.0 MPa)
Solid Fraction	85 ± 0.05%

^1^ In-process (HME) quality material attributes of the intermediate product ITZASD containing 30% ITZ and 70% Kollidon VA64.

**Table 2:** Risk assessment using Failure Mode Effect Analysis (FMEA) for ITZ ASD manufacture and ASD tablet development.

Index Number	Risk Area	Potential Failure Mode	Potential Failure Effects	S	O	D	RPN *	Mitigations	Revised Ranking			
									S	O	D	RPN
1	API	Concentration, PSD, morphology, impurity	Inaccurate dose	7	4	2	56	Standard operation procedure for dispensing API accurately. Calibration of balance. Check certificate of analysis. Verification chemical identity and physical chemical properties	7	4	1	28
2	Polymer matrix	Residual water	API crystallisation	7	4	2	56	Perform thermogravimetric analysis to determine water content. Control storage conditions (temperature and humidity)	7	1	1	7
3	Excipients	Out of specifications	Impact on critical quality attributes of product	7	2	2	28	Check supplier’s certificate of analysis. If necessary, perform analytical tests.	7	1	1	7
8	Mill	Particle size distribution (PSD)	Impact on dissolution profile	10	7	4	280	Determine the particle size distribution	10	4	1	40
4	Blender	Time, speed, type	Content uniformity	10	4	4	160	Perform content uniformity validation to identify optimum time and speed of mixing. Selected blender type, Turbula	10	4	1	40
5	STYL’One Nano Hopper	Powder flow, calculated die fill level not achieved	Content uniformity, weight variation	7	5	4	140	Angle of repose to determine flow properties or increase hopper vibrations	7	4	1	28
9	Compaction	Powder flow, PSD, formulation excipients, machine calibration, profile type, speed, compression pressure, tablet size and weight	Tensile Strength, solid fraction, Disintegration and Dissolution	10	7	4	280	Measure manufacturability profile. Perform DoE to optimise formulation and compaction conditions	10	4	2	80
10	Personnel	Sample labelling, data collection, lab skills and competence	Product quality	7	4	1	28	Training programme aided by the use of SOPs	7	1	1	7
11	Storage	Sample change with time	Variability on product quality attributes	7	4	1	28	Keep samples in controlled storage conditions for temperature and humidity	7	1	1	7

* S = Severity, O = Occurrence, D = Detectability, RPN = Risk priority number. Risk code of RPN by colour: Red = high, Yellow = medium, Green = low.

**Table 3:** ASD ITZ tablet formulations containing salts.

Component	Functionality	%
ITZ ASD	Active	44.4
Avicel^®^ pH102	Diluent	10
Tablettose^®^ 70	Diluent	27.6
Kollidon^®^-CL-SF	Disintegrant	7.5
Inorganic Salt	Soluble Adjuvant	10
Magnesium Stearate	Lubricant	0.5

**Table 4:** Mixture design and ternary plot for ITZ ASD tablets formulations.

Component (%)	Formulation No.							Ternary Diagram
	1	2	3	4	5	6	7	
								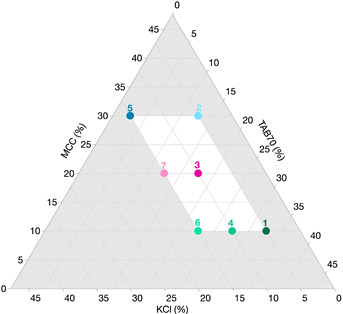
ITZ ASD			44.4					
							
KCl	5	5	10	10	15	15	15	
							
MCC	10	30	20	10	30	10	20	
							
TAB70	32.6	12.6	17.6	27.6	2.6	22.6	12.6	
							
KOL-CL-SF			7.5					
							
MgSt			0.5					

**Table 5:** Factors and responses for the mixture design of round and oblong tablets.

				Round			Oblong		
Formulation No.	KCl (%)	MCC (%)	TAB70 (%)	Tensile Strength (MPa)	Solid Fraction (%)	Disintegration Time (min)	Tensile Strength (MPa)	Solid Fraction (%)	Disintegration Time (min)
1	5	10	32.6	2.0	89.31	2.09	1.6	82.71	2.08
2	5	30	12.6	3.7	89.84	20	3	85.87	10.37
3	10	20	17.6	2.7	89.22	9.76	2.5	85.16	0.69
4	10	10	27.6	1.8	87.17	2.53	1.5	83.35	0.66
5	15	30	2.6	2.9	85.89	4.39	2.7	80.70	1.16
6	15	10	22.6	1.7	89.18	1.73	1.6	82.44	0.54
7	15	20	12.6	2.1	81.66	0.76	2.3	84.95	0.35

**Table 6:** Statistical analysis of the mixture design formulation data.

	Tensile Strength (MPa)		Disintegration Time (min)	
	Round	Oblong	Round	Oblong
R^2^ Adj	0.95	0.93	0.63	0.60
F Ratio	54.23	38.23	6.19	5.42
Prob > F	0.0013 *	0.0025 *	0.0596	0.0727

* *p* < 0.05.

**Table 7:** CQAs responses for tablets of ITZ-KOL-ASD(±KCl) and ITZ-KOL-PM(+KCl).

CQAs	ITZ-KOL-ASD (+KCl) (%)	ITZ-KOL-ASD (-KCl) (%)	ITZ-KOL-PM (+KCl) (%)
Tensile Strength (MPa)	2	3.2	4.4
Solid Fraction (%)	91	89	90
Disintegration time (s)	4	>60	8
Friability (%)	0.23	0.26	0.25
Dissolution @30 min	100.0	21.0	7.0

## Data Availability

The data presented in this study are available upon request from the corresponding author.
